# Prediction of medication-related osteonecrosis of the jaws using machine learning methods from *estrogen receptor 1* polymorphisms and clinical information

**DOI:** 10.3389/fmed.2023.1140620

**Published:** 2023-06-21

**Authors:** Seo-Yong Choi, Jin-Woo Kim, Sang-Hyeon Oh, Seunghyun Cheon, Jeong Yee, Sun-Jong Kim, Hye Sun Gwak, Jee-Eun Chung

**Affiliations:** ^1^College of Pharmacy and Institute of Pharmaceutical Science and Technology, Hanyang University, Ansan, Republic of Korea; ^2^Department of Oral and Maxillofacial Surgery, School of Medicine, Ewha Womans University, Seoul, Republic of Korea; ^3^College of Pharmacy and Graduate School of Pharmaceutical Sciences, Ewha Womans University, Seoul, Republic of Korea

**Keywords:** genetic polymorphism, *ESR1*, medication-related osteonecrosis of the jaw, MRONJ, machine learning, risk score

## Abstract

**Objective:**

The purpose of this study was to evaluate the effect of *estrogen receptor 1* (*ESR1*) polymorphisms on the development of medication-related osteonecrosis of the jaws (MRONJ) in women with osteoporosis.

**Methods:**

A total of 125 patients taking bisphosphonates was evaluated the relationship between MRONJ occurrence and single nucleotide polymorphisms (SNPs) of *ESR1*. Clinical information was collected, including current age, treatment duration, and comorbidity. Univariate and Multivariable regression analyzes were performed to evaluate the independent predictive factors for MRONJ occurrence. Predictive models were constructed using machine learning methods such as Lasso regression, Random forest (RF), and Support vector machine (SVM). The area under the receiver-operating curve (AUROC) was used to evaluate the performance of a binary classifier.

**Result:**

Two SNPs of *ESR1* (rs4870056 and rs78177662) were significantly associated with MRONJ development. Patients with variant allele (A) of rs4870056 showed 2.45 times (95% CI, 1.03–5.87) the odds of MRONJ occurrence compared to those with wild-type homozygote (GG) after adjusting covariates. Additionally, carriers with variant allele (T) of rs78177662 had higher odds than those with wild-type homozygote (CC) (adjusted odds ratio (aOR), 2.64, 95% CI, 1.00–6.94). Among demographic variables, age ≥ 72 years (aOR, 3.98, 95% CI, 1.60–9.87) and bisphosphonate exposure ≥48 months (aOR, 3.16, 95% CI, 1.26–7.93) were also significant risk factors for MRONJ occurrence. AUROC values of machine learning methods ranged between 0.756–0.806 in the study.

**Conclusion:**

Our study showed that the MRONJ occurrence was associated with *ESR1* polymorphisms in osteoporotic women.

## 1. Introduction

Bisphosphonates (BPs) are commonly used agents with anti-resorptive actions for skeletal protection in osteoporosis, multiple myeloma, and cancer bone metastasis (1, 2). Despite their clinical usefulness, they have some limitations because of safety concerns. In 2003, Marx first reported 36 cases of jaw bone necrosis that appeared to be related to BPs (3). Since then, similar cases have been reported, which are commonly called bisphosphonate-related osteonecrosis of the jaws (4, 5). In 2014, the American Association of Oral and Maxillofacial Surgeons (AAOMS) changed to the broad term “Medication-Related Osteonecrosis of the Jaws” (MRONJ) (6), as it is not only caused by bisphosphonates, but also by other anti-resorptive and anti-angiogenic drugs (7).

As case reports of MRONJ increased, a large number of researches have been performed to find the mechanisms, and its pathogenesis presumed disturbed bone remodeling, inflammation or infection, altered immunity, and angiogenesis inhibition (8–10). It was reported that 63.7% of MRONJ cases are related to tooth extraction (11). Therefore, the influence of medications including BPs on bone remodeling has been extensively studied (12). Bone remodeling consists of three consecutive phases: resorption, reversal, and formation (13, 14). During the healing period of tooth extraction, especially in the early stages, osteoclastic resorption of the bone is considered the first important feature to repair from bone remodeling (15).

Estrogen is a well-known regulator of bone turnover and acts by binding estrogen receptors (ESR) (16–18). For bone remodeling, it has been reported that *estrogen receptor 1* (*ESR1*, ERα) affects both osteoblasts and osteoclasts, and ESR2 (ERβ) mainly affects osteoblasts (19–21). *ESR1* mediates the direct effects of estrogen on osteoclasts. For the effects on osteoblast, estrogen binds *ESR1* of osteoblast progenitors and leads to attenuated bone resorption at the endo-cortical surface. Additionally, the *ESR1* of osteoblast progenitors stimulates periosteal bone development in response to mechanical strain, independently of estrogens (22, 23).

*ESR1* is located on chromosome 6q25. Genetic screening of the *ESR1* has found several meaningful polymorphic sites. Among polymorphisms, rs2234693 (PvuII) has been studied most actively, especially for its effects on bone mineral density and fracture risk, although results are conflicting (24, 25). Given the wide role of *ESR1* in bone turnover, *ESR1* polymorphisms in postmenopausal women could also be considered in the occurrence of MRONJ. However, it has not been studied much possibly because of involving complex processes (25).

Moreover, many of MRONJ studies have been carried out in oncology patients with solid tumors and multiple myeloma (24, 26). In addition, most such studies enrolled healthy controls without taking a BP (27, 28).

Therefore, the aim of the present study is to investigate the association between *ESR1* polymorphism and MRONJ occurrence in osteoporosis patients taking BPs. Additionally, to quantify the risk of MRONJ, this study attempts to construct a predictive model to apply several machine learning techniques.

## 2. Methods

### 2.1. Patients and data collection

This study was an analysis of prospectively collected saliva samples from January 2014 to December 2018 at EWHA Womans University Mokdong Hospital. The detailed explanation of the study patients have already been provided in our previous paper (29). Briefly, all participants with current or previous BP use were diagnosed with osteoporosis by a physician. MRONJ was diagnosed by oral surgeons in accordance with AAOMS’ guidelines. Case group was identified as those who developed MRONJ, and control group was defined as those who had not developed MRONJ after dentoalveolar surgery. Clinical information was collected during the patients’ outpatient clinic visits. The collected clinical information included patients’ age, comorbidities, and duration of BP use.

The study protocol was approved by ethics committee of EWHA Womans University Mokdong Hospital Institutional Review Board (IRB number: 14–13-01), and written informed consent was obtained from all patients before their participation in the study.

### 2.2. Genotyping

Genomic DNA was extracted from saliva samples collected using the tube format (OG300) of the Oragene®•DNA Self-Collection Kit (DNAgenotek, Ontario, Canada), according to manufacturer’s instructions. SNPs of *ESR1* were selected based on other studies and genetic information obtained from the SNP database of the National Center for Biotechnology Information (dbSNP) (25, 30–32). For the selection of *ESR1* SNPs with minor allele frequency (MAF) of ≥20% in Japanese and Han Chinese populations, the following two methods were used: genetic information was obtained from Haploreg v4.1 and the tagger function was implemented in Haploview v4.2 program (33). Linkage disequilibrium blocks were constructed following the D′ method (34). One SNP in 5’UTR (rs78177662), eight intronic SNPs (rs827420, rs4870056, rs6912184, rs722208, rs851967, rs17081716, rs2175898, rs9371226) were selected.

### 2.3. *In silico* analysis

Several computational tools were used to predict the possible effects of given variants on splicing. ESEfinder, SpliceAid2, and EX-SKIP were used to evaluate alternations of the splicing factor-binding site pattern caused by the given point mutation (35–37). The default threshold value was used and a score for a sequence above the threshold was considered to be potentially significant. Also, most splicing factors bind short (4–10 nucleotide) and degenerate sequences (38). For SNP sequence information, a total of 15 nucleotides was used for analysis using dbSNP.

### 2.4. Statistical analysis and machine learning methods

The chi-square and Student’s t-test were used to compare categorical and continuous variables between case and control groups, respectively. Multivariable logistic regression analysis was used to examine independent risk factors for MRONJ. The clinical and genetic variables selected were those with *p* < 0.2 on univariate analysis. Variables were entered by stepwise selection for *p* < 0.1 and were removed for *p* > 0.05. Odds ratios (ORs) and adjusted odds ratios (aORs) were calculated from univariate and multivariable analyzes, respectively. All statistical tests were conducted with a two-tailed alpha of 0.05. Machine learning models were run on each dataset in 5 iterations of tenfold cross-validation on hyperparameter tuning to enhance the effectiveness of verification. Train and verification data were divided into 0.75 and 0.25 of the total. The importance of features of the model was also confirmed. Discrimination of the model was assessed by an analysis of the area under receiver operating curve (AUROC) and its 95% confidence interval (CI) of each model (39).

A risk scoring system was developed from the multivariable and machine learning analyzes. We randomly divided the data by a ratio of 75:25. To obtain the risk score, each coefficient from the logistic regression model was divided by the smallest one and rounded to two decimal places.

Statistical Package for Social Sciences Version 20.0 for Windows (SPSS, Chicago, IL, United States,^1^) was used for all analyzes. Machine learning algorithms were constructed using R software version 3.6.0 (RFoundation for Statistical Computing, Vienna, Austria,^2^).

## 3. Results

Initially, a total of 149 patients were screened and enrolled in this study. Twenty who had additional indications other than osteoporosis, two who had missed clinical information, and two men were excluded. A total of 125 patients were included in the final analysis and 58 (46.4%) developed MRONJ after dental procedures. Table 1 shows the demographic and clinical characteristics of the study population stratified by MRONJ occurrence. The mean age was 72.9 ± 9.4, and the most frequent comorbidity was hypertension. Sixty four patients had a history of hypertension, which showed statistical significance in relation to MRONJ occurrence (*p* = 0.024). The proportion of patients who had taken BPs for 48 months or longer was higher in the case group than the control group (*p* = 0.003).

**Table 1:** Demographic characteristics.

Characteristics	Case (*n* = 58)	Control (*n* = 67)	*P*
Age			0.001
<72	13 (22.4)	35 (52.2)	
≥72	45 (77.6)	32 (47.8)	
Comorbidity			
Hypertension	36 (62.1)	28 (41.8)	0.024
Diabetes mellitus	18 (31.0)	16 (23.9)	0.370
Cardiovascular disease	8 (13.8)	8 (11.9)	0.757
Rheumatoid arthritis	7 (12.1)	2 (3.0)	0.080
Thyroid disease	4 (6.9)	2 (3.0)	0.415
Kidney disease	2 (3.4)	3 (4.5)	1.000
Liver disease	0 (0)	2 (3.0)	0.499
Treatment duration			0.003
<48	28 (48.3)	46 (68.7)	
≥48	27 (46.6)	13 (19.4)	
ND	3 (5.2)	8(11.9)	

ND, not determined.

The effects of 9 SNPs of *ESR1* on the occurrence of MRONJ were evaluated (Table 2). Minor allele frequencies (MAFs) in our study population ranged from 28.9 to 48.8%. In univariate analysis, rs827420 (G > A) was significantly associated with the occurrence of MRONJ. Carriers with variant A allele of rs827420 had a higher incidence rate of MRONJ than did those with wild homozygote carriers (*p* = 0.02). The polymorphisms of rs4870056 (G > A), rs78177662 (C > T), rs851967 (C > T) and rs9371226 (G > T) reached marginal significance (Table 2).

**Table 2:** Association of genotypes with medication-related osteonecrosis of jaws.

SNP	Allele change	Minor allele frequency	dbSNP func annot	Grouped genotypes	Case (*n* = 58)	Control (*n* = 67)	*p*	HWE
rs827420	G > A	0.435	Intronic	GG	13	28	0.020	0.684
				GA,AA	45	38		
rs4870056	G > A	0.309	Intronic	GG	21	36	0.051	0.567
				GA,AA	36	30		
rs6912184	G > A	0.468	Intronic	GG	14	10	0.196	0.280
				GA,AA	44	57		
rs78177662	C > T	0.456	5’UTR	CC	45	25	0.051	0.949
				CT,TT	12	42		
rs722208	A > G	0.384	Intronic	AA	7	14	0.193	0.408
				AG,GG	51	53		
rs851967	C > T	0.488	Intronic	CC	9	20	0.062	0.883
				CT,TT	49	47		
rs17081716	A > G	0.289	Intronic	AA	23	36	0.117	0.210
				AG,GG	34	30		
rs2175898	C > T	0.468	Intronic	CT,CC	16	55	0.198	0.920
				TT	42	12		
rs9371226	G > T	0.296	Intronic	GT,GG	54	56	0.059	0.254
				TT	3	10		

For the multivariable analysis, the SNP of rs827420 was excluded because of multi-collinearity with age variable in our population. After adjusting covariates, carriers with variant allele of rs4870056 had approximately 2.45 times (95% CI 1.03–5.87) the odds of MRONJ occurrence compared to those with wild-type homozygotes (GG). Carriers with variant T allele of rs78177662 had 2.64 times higher incidence of MRONJ than did those with other genotypes. The age over 72 years (aOR 3.98, 95% CI 1.60–9.87) and treatment duration over 48 months (aOR 3.16, 95% CI 1.26–7.93) were both significant clinical predictors of MRONJ development (Table 3).

**Table 3:** Multivariable analysis to identify predictors of medication-related osteonecrosis of jaws.

Variables	Crude odds ratio (95% CI)	Adjusted odds ratio (95% CI)
Age ≥ 72 (years)	3.79 (1.73–8.27)^**^	3.98 (1.60–9.87)^**^
Treatment duration ≥48 (months)	3.40 (1.52–7.68)^**^	3.16 (1.26–7.93)^*^
*ESR1* rs4870056 GA/AA	2.06 (1.00–4.24)	2.45 (1.03–5.87)^*^
*ESR1* rs78177662 CT/TT	2.23 (1.00–5.00)	2.64 (1.00–6.94)^*^

Logistic regression analysis with backward elimination was carried out with variables such as age ≥ 72, treatment duration ≥ 48, rheumatoid arthritis, hypertension, rs4870056, rs6912184, rs78177662, and rs2175898.

^*^p < 0.05, ^**^p < 0.01.

Analysis of SNP rs4870056 with ESEfinder 3.0, which detects exonic splicing enhancer (ESE) for the SR protein, showed that this mutation decreased the score of SF2/ASF binding from −0.308 to −2.372 (threshold: 1.956). The polymorphism of rs78177662, which located on 5’UTR also associated with the change of exonic splicing silencer (ESS)/ESE ratio in EX-SKIP program, showed that this mutation increased the ESS/ESE ratio from 0.00 to 2.00 (Table 4).

**Table 4:** *In silico* analysis.

SNP ID	Program version	Nucleotide change	Variation	Genomic position	Site change (score, total)
rs4870056	ESEfinder3.0				SRSF2(2,6) → SRSF2(2.0)
	EX-SKIP	G > A	Intron Variant	6:151841092 (GRCh38)	ESS(16), ESE(8) → ESS(15), ESE(6)
	SpliceAid2				CUG-BP1(5), ETR-3(5), Nova-1(5) → ETR-3(5)
rs78177662	ESEfinder3.0	C > T			SRSF1(1.6), SRSF2(1.0) → SRSF1(−1.4), SRSF2(0.8)
	EX-SKIP		5 Prime UTR Variant	6:151654430 (GRCh38)	ESS(0), ESE(1) → ESS(2), ESE(0)
	SpliceAid2				NA

NA, not available.

**Figure 1:**
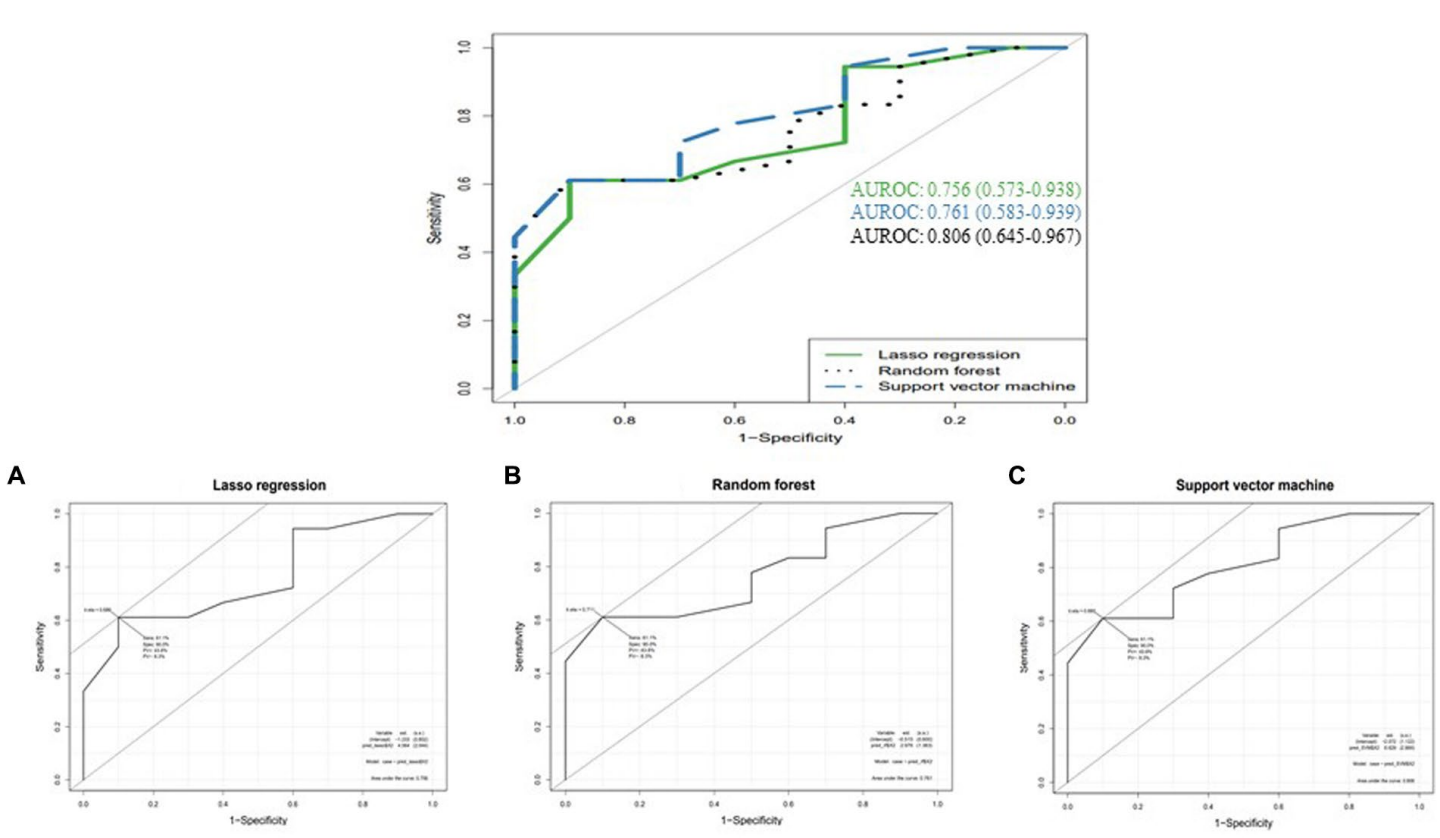
Comparison of AUROCs between machine learning methods **(A)** Lasso regression, **(B)** Random forest, and **(C)** Support vector machine.

The top four important features of machine learning methods were the highest at aged over 72 years, followed by duration over 48 months from Random forest (RF) and Support vector machine (SVM) and rs4870056 from Lasso regression (Table 5). The AUROC values (mean, 95% CI) using ten-fold cross-validated Lasso regression, SVM and RF models were 0.756 (0.573–0.938), 0.761 (0.583–0.939), and 0.806 (0.645–0.967), respectively (Figure 1). The hyperparameters and R code that we used are shown in Supplementary file S1.

**Table 5:** The top four important features of machine learning methods.

Rank	Machine learning methods		
	Lasso regression	Random forest	Support vector machine
1	Age ≥ 72	Age ≥ 72	Age ≥ 72
2	rs4870056	Treatment duration ≥48	Treatment duration ≥48
3	Treatment duration ≥48	rs4870056	rs4870056
4	rs78177662	rs78177662	rs78177662

**Figure 2:**
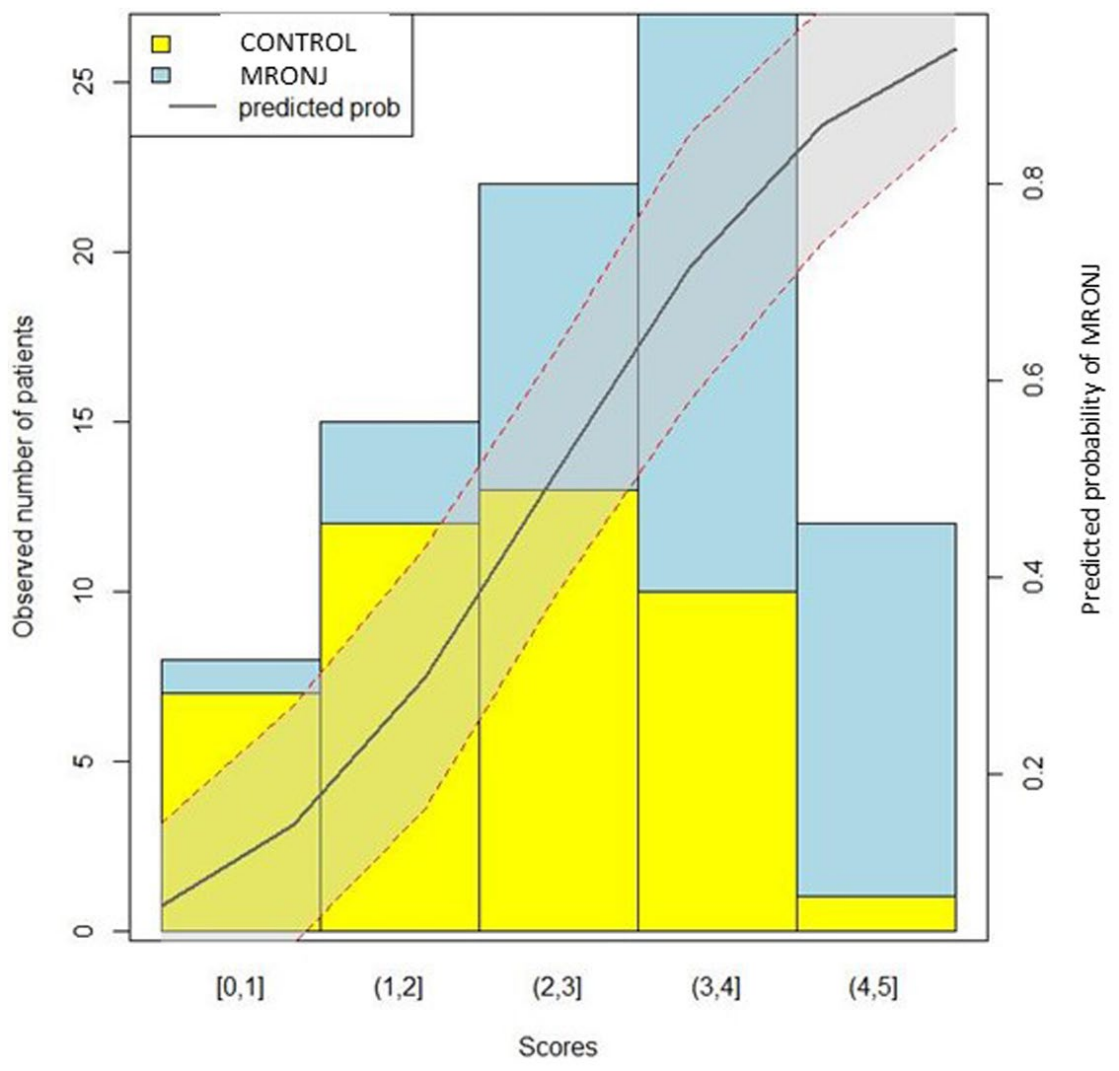
Risk scores vs. probability of MRONJ.

Patients with 0, 1–2, 2–3, 3–4, and 4–5 points on the training set showed approximately 0, 20, 41, 63, and 92% risk of MRONJ, respectively. The respective value of the validation set was 0, 25, 36, 71, and 100%. The logistic regression curve by mapping the scores to risk scores is presented in Figure 2.

## 4. Discussion

This study showed that *ESR1* polymorphisms affected MRONJ development in postmenopausal women. Variant A allele of rs4870056 and variant T allele of rs78177662 increased the risk of MRONJ by 2.45 fold and 2.64 fold, respectively. Patients aged 72 and older, and treatment duration over 48 months had an increased risk of MRONJ by 3.98 fold and 3.16, respectively. Machine learning analyzes indicated good performance (higher than 0.7) of the constructed model.

Previous pharmacogenomic studies have shown the relationships of some genes with the occurrence of MRONJ using population-based or healthy controls without taking BPs (27, 28, 40). In contrast, our group recently reported that *VEGFA* polymorphisms, involved in the modulation of angiogenesis, could affect the development of MRONJ, comparing osteoporotic controls taking BPs (29). Furthermore, it may essential in our study population to evaluate the impact of genetic variants of *ESR1*, which encodes estrogen receptor that binds estrogen playing a crucial role in bone turnover.

BPs suppress mandibular bone remodeling in experimental animals (41). It has been proposed that reduced jaw bone turnover may impede the healing process even for a mild injury such as a dental extraction (42). Estrogen deprivation in postmenopausal women results in accelerated bone resorption and remodeling (43–45). It leads to reduce bone turnover. Another potential mechanism is that *ESR1* affects the activation of the insulin-like growth factor 1 (IGF-1) signaling pathway, mainly involving anti-inflammatory reactions and re-epithelialization (46, 47). IGF-1 can promote wound recovery in estrogen-deficient animal models. However, it showed that IGF-1 does not promote healing and increases inflammatory responses in *ESR1*-null mice (48). In patients taking BPs, it suggested that *ESR1* polymorphism leads to decreased bone turnover and poor wound healing and may contribute to the development of MRONJ (25).

The SNP of rs4870056, located in *ESR1* intron, this SNP has been studied in several other clinical fields. It reported that rs4870056 was associated with cardiovascular disease risk during postmenopausal hormone therapy (49, 50). Checking the linkage disequilibrium, rs4870056 appeared to have a strong relationship with rs2234693 (*Pvull*), which is currently one of the most widely studied SNPs among *ESR1* SNPs. From studies using rs2234693, it was explained that the SNP might contribute to determining of bone mineral density and fracture in postmenopausal women (32, 51, 52). In addition, *in silico* analysis showed that splicing factors tended to decrease due to changes in motifs in the mutant sequence. The polymorphism of rs78177662 is located at 5’UTR, but the current study has not revealed its function. It could only be inferred from the *in silico* analysis.

Recently, several studies reported the influence of gene polymorphism on the development of disease and drug-related adverse reactions applying machine learning (53–55). Some machine learning methods such as RF, SVM, and decision trees classification were used, and the performance of each model was compared with AUROC values (53). In this study, the purpose of generating models is to provide a warning for and screen a group with an increased risk for MRONJ occurrence. Applying machine learning methods can improve the performance in predicting (55, 56). To evaluate AUROC values, all our machine learning models employed performed well. Lasso regularization shrinks regression coefficients toward zero, effectively selecting significant predictors and improving the interpretability of the model (57). As a result of regulating variable weights with lasso, 0.756 of AUROC value was obtained. Using the bagging technique, RF can generate multiple versions of predictors, and use them to obtain aggregated predictors (58). An AUROC value was 0.761 from the test set. As another classification model is the maximization of separating margin, and the AUROC value of SVM was 0.806 after setting the optimal cost and gamma using the tune function (59).

Risk assessment systems, including biomarkers and risk factors, are helpful for rapid clinical decision-making. Risk scoring systems for adverse drug reactions (ADR), such as the GerontoNet ADR risk score, have been developed (60). Therefore, this study developed the risk scoring system for MRONJ occurrence in addition to previous work. The constructed risk scoring system may be helpful to screen the high-risk group for MRONJ occurrence, and further studies are needed to improve its generalizability.

This study has several limitations due to the study design and sample size. The mechanism that polymorphisms of *ESR1* affect osteonecrosis could not be investigated. However, we attempted to understand the roles of SNP through *in silico* analysis. The study’s strength is that it collected data from MRONJ patients with osteoporosis and created predictive models and a risk-scoring system through machine learning methods. This study showed that *ESR1* polymorphism in female patients with estrogen deficiency was associated with medication-related osteonecrosis.

## 5. Conclusion

Our study showed that MRONJ development was associated with *ESR1* gene polymorphism in osteoporotic women. Predictive modeling created through machine learning techniques showed good performances. As a result, from the perspective of bone remodeling, it suggests the possibility of predictive diagnosis.

## Data availability statement

The original contributions presented in the study are publicly available. This data can be found here: https://www.ncbi.nlm.nih.gov/SNP/snp_viewBatch.cgi?sbid=1063487.

## Ethics statement

The studies involving human participants were reviewed and approved by EWHA Womans University Mokdong Hospital. The patients/participants provided their written informed consent to participate in this study.

## Author contributions

HG and J-EC: conceptualization. J-WK and S-JK: data curation. S-YC, S-HO, and SC: formal analysis. J-WK and J-EC: funding acquisition. JY, HG, and J-EC: methodology. J-WK, HG, and J-EC: supervision. S-YC, HG, and J-EC: writing–original draft. S-YC, J-WK, S-HO, SC, JY, S-JK, HG, and J-EC: writing–review and editing. All authors contributed to the article and approved the submitted version.

## Funding

This work was supported by a grant of the Korea Health Technology R&D Project through the Korea Health Industry Development Institute (KHIDI) funded by the Ministry of Health & Welfare, Republic of Korea (HI22C1377), and the National Research Foundation of Korea (NRF) grants funded by the Ministry of Education (2020R1A2C4001842 and 2022R1F1A1075439).

## Conflict of interest

The authors declare that the research was conducted in the absence of any commercial or financial relationships that could be construed as a potential conflict of interest.

## Publisher’s note

All claims expressed in this article are solely those of the authors and do not necessarily represent those of their affiliated organizations, or those of the publisher, the editors and the reviewers. Any product that may be evaluated in this article, or claim that may be made by its manufacturer, is not guaranteed or endorsed by the publisher.

## Supplementary material

The Supplementary material for this article can be found online at: https://www.frontiersin.org/articles/10.3389/fmed.2023.1140620/full#supplementary-material

## Footnotes

^1^ https://www.ibm.com/spss

^2^ https://www.r-project.org
