# Supplementary material for: Prediction of medication-related osteonecrosis of the jaws using machine learning methods from estrogen receptor 1 polymorphisms and clinical information
Source: Front Med (Lausanne). 2023 Jun 21;10:1140620. doi: 10.3389/fmed.2023.1140620 (PMC10321771; doi:10.3389/fmed.2023.1140620)
Supplement: Supplementary file 1 [file Data_Sheet_1.PDF]

Supplementary 1. Hyperparameters for machine learning methods

Model Type	R Package	Hyperparameter	Value
Logistic regression	caret/glmnet	-	-
Lasso	caret/Lasso	lamdba	0.03
		alpha	0.1
Random forest	caret/rf	mtry	2
		ntree	500
Support vector machine	caret/svmRadialSigma	C	0.5
		sigma	0.07

```
#Machine learning
ESR = read.csv("ESR_machine.csv", sep = ",", header = T, fill = T, na.strings = " ")
for(n_col in 1:5){
  ESR[,n_col] = as.factor(ESR[,n_col])
}
str(ESR)
set.seed(27)
indexTrain <- sample(1:nrow(ESR), round(nrow(ESR)*.75))
train <- ESR[indexTrain,]
vaildation <- ESR[-indexTrain,]
fitControl <- trainControl(method = "repeatedcv", number= 10, repeats = 5,
savePredictions = T,classProbs = TRUE, verboseIter = TRUE)
#LASSO
Lasso_fit <- train(case~., data=train, method = "glmnet",
                   trControl = fitControl, verbose = F)
pred_lasso <- predict(Lasso_fit, newdata = vaildation, type = "prob")
Lasso_pred <- roc(vaildation$case, pred_lasso$X2, ci =T, direction = "<")
Lasso_pred
plot.roc(Lasso_pred, print.auc = T)
plot(varImp(Lasso_fit,scale=F), main= "Lasso Imp:  10 fold CV")
#RF
rf_fit <- train(case ~ rs4870056 + rs78177662 + age_72 + duration_48 , data=train, method
= "rf",ntree = 500,
                trControl = fitControl, verbose = F)
```

```
pred_rf <- predict(rf_fit, newdata = vaildation, type = "prob")

RF_pred <- roc(vaildation$case, pred_rf$X2, ci =T, direction = "<")

RF_pred

plot.roc(RF_pred,add=TRUE, print.auc=TRUE)

plot(varImp(rf_fit,scale=F), main= "var Imp: RF 10 fold CV")

#SVM

SVM_fit <- train(case ~ ., data=train, method = "svmRadialSigma",

                    trControl = fitControl, verbose = F)

pred_SVM <- predict(SVM_fit, newdata = vaildation, type = "prob")

SVM_pred <- roc(vaildation$case, pred_SVM$X2, ci =T, direction = "<")

SVM_pred

plot.roc(SVM_pred, print.auc = TRUE)

plot(varImp(SVM_fit,scale=F), main= "svm Imp:   10 fold CV")

ROC(form= case~pred_lasso$X2, data = vaildation, plot = "ROC",cex.lab = 1.5, main = "(A)
Lasso regression", cex.main =2)

ROC(form= case~pred_rf$X2, data = vaildation, plot = "ROC",cex.lab = 1.5, main = "(B)
Random forest", cex.main =2)

ROC(form= case~pred_SVM$X2, data = vaildation, plot = "ROC",cex.lab = 1.5, main = "(C)
Support vector machine", cex.main =2)

#Comparison of ROC

plot.roc(Lasso_pred, col= "#4daf4a", lty=1, lwd= 6, auc.polygon = FALSE,

          max.auc.polygon = FALSE, print.thres = FALSE, cex.lab = 1.2, xlab = "1-
Specificity", ylab="Sensitivity")

plot.roc(RF_pred,add=TRUE, col= "black", lty=3, lwd= 6)
```

```
plot.roc(SVM_pred, add=TRUE, col= "#377eb8", lty=5, lwd= 6)
legend("bottomright", legend=c("Lasso regression", "Random forest","Support vector machine"), col =c("#4daf4a","black","#377eb8"), lty=c(1,3,5), lwd=3, cex =1.2)
ROC(form= case~pred_lasso$X2, data = vaildation, plot = "ROC",cex.lab = 1.5, main = "(A) Lasso regression", cex.main =2)
ROC(form= case~pred_rf$X2, data = vaildation, plot = "ROC",cex.lab = 1.5, main = "(B) Random forest", cex.main =2)
ROC(form= case~pred_SVM$X2, data = vaildation, plot = "ROC",cex.lab = 1.5, main = "(C) Support vector machine", cex.main =2)

#scoring
df = read.csv("ESR_SCORE.csv", sep = ",", header = T, fill = T, na.strings = " ")
ESR.F<-data.frame(c(1:111))
for (var in c("age_72","duration_48","rs4870056","rs78177662","case")){
  ESR.F <-cbind(ESR.F,df[,var])
}
colnames(ESR.F) = c("id","age_72","duration_48","rs4870056","rs78177662","case")
mod <-glm(case~age_72+duration_48+rs4870056+rs78177662
          ,family=binomial,data=ESR.F )
coef(mod)
summary(mod)
score <-round(coef(mod),1)
score<-round(score[2:5]/0.9, 1)
for(var in names(score)){
```

```
  ESR.F[,paste(var,"points",sep=".")] <-as.numeric(NA)
}
for(var in names(score)){
  score.var<-score[var]
  names(score.var) <-sub(var,"",names(score.var))
  for (i in 1:(length(ESR.F[,var])-1)){
    ESR.F[,paste(var,"points",sep=".")]<-ifelse(
      ESR.F[,var]==1&
        is.na(ESR.F[,paste(var,"points",sep=".")]),
      score.var[i],
      ESR.F[,paste(var,"points",sep=".")]
    )
  }


}
for(var in names(score)){
  ESR.F[,paste(var,"points",sep=".")]<-ifelse(
    is.na(ESR.F[,paste(var,"points",sep=".")]),
    0,ESR.F[,paste(var,"points",sep=".")]
  )
}
ESR.F$score<-rowSums(ESR.F[,grepl("\\.+points",names(ESR.F))])
head(ESR.F[])
ESR.F$dataset = sample(x=c("train", "validate"), size = 111, replace = TRUE, prob = c(0.75,
```

```
0.25))
train = ESR.F[ESR.F$dataset=="train", ]
validation = ESR.F[ESR.F$dataset=="validate", ]
glmod <-glm(case~score,
            train,
            family="binomial")
newx<-seq(min(train$score),max(train$score)+1)
newx
prd<- predict(glmod,
              newdata=data.frame(score=newx),
              type="response",
              se.fit=T)
count <-as.matrix(table(cut(train$score,
                            breaks=seq(min(train$score),
                                       max(train$score)),
                            include.lowest = T),
                        train$case))
t(count)
library(ggplot2)
par(mar=c(5,5,3,10))
barplot(t(count),
        main="Scores versus probability of Case",
        xlab="Scores",
        ylab="Observed number of patients",
```

```
             space=0,
             col=c("yellow","lightblue"))
legend("topleft",fill=c("yellow","lightblue",NA),
            lty=c(NA,NA,1), lwd=c(NA,NA,0.5),
            legend=c("CONTROL","BRONJ",
                         "predicted prob"),
            col=c("black"),
            border=c("black","black",NA))
par(new=TRUE)
plot(prd$fit~newx,
        type="l",col="black",
        lwd=2,xaxt="n",yaxt="n",
        xlab="",ylab="")
polygon(c(rev(newx),newx),
             c(rev(prd$fit+1.96*prd$se.fit),
                prd$fit-1.96*prd$se.fit),
             col=adjustcolor("grey80",alpha=0.5),
             border=NA)
lines(newx,prd$fit+1.96*prd$se.fit,
          lty="dashed", col="red")
lines(newx,prd$fit-1.96*prd$se.fit,
          lty="dashed",col="red")
mtext("predicted probability of case",
          side=4, line=3)
```
